# Targeting Macrophages: Friends or Foes in Disease?

**DOI:** 10.3389/fphar.2019.01255

**Published:** 2019-10-23

**Authors:** Juan A. Ardura, Gorjana Rackov, Elena Izquierdo, Veronica Alonso, Arancha R. Gortazar, Maria M. Escribese

**Affiliations:** ^1^Department of Basic Medical Sciences, Faculty of Medicine, San Pablo CEU University, Madrid, Spain; ^2^IMDEA Nanoscience Institute, Madrid, Spain; ^3^Fundación de Investigación HM Hospitales, Madrid, Spain; ^4^Department I for Internal Medicine and CECAD, University Hospital of Cologne, Cologne, Germany

**Keywords:** macrophages, metastasis, osteomacs, arthritis (including rheumatoid arthritis), glioblastoma

## Abstract

Macrophages occupy a prominent position during immune responses. They are considered the final effectors of any given immune response since they can be activated by a wide range of surface ligands and cytokines to acquire a continuum of functional states. Macrophages are involved in tissue homeostasis and in the promotion or resolution of inflammatory responses, causing tissue damage or helping in tissue repair. Knowledge in macrophage polarization has significantly increased in the last decade. Biomarkers, functions, and metabolic states associated with macrophage polarization status have been defined both in murine and human models. Moreover, a large body of evidence demonstrated that macrophage status is a dynamic process that can be modified. Macrophages orchestrate virtually all major diseases—sepsis, infection, chronic inflammatory diseases (rheumatoid arthritis), neurodegenerative disease, and cancer—and thus they represent attractive therapeutic targets. In fact, the possibility to “reprogram” macrophage status is considered as a promising strategy for designing novel therapies. Here, we will review the role of different tissue macrophage populations in the instauration and progression of inflammatory and non-inflammatory pathologies, as exemplified by rheumatoid arthritis, osteoporosis, glioblastoma, and tumor metastasis. We will analyze: 1) the potential as therapeutic targets of recently described macrophage populations, such as osteomacs, reported to play an important role in bone formation and homeostasis or metastasis-associated macrophages (MAMs), key players in the generation of premetastatic niche; 2) the current and potential future approaches to target monocytes/macrophages and their inflammation-causing products in rheumatoid arthritis; and 3) the development of novel intervention strategies using oncolytic viruses, immunomodulatory agents, and checkpoint inhibitors aiming to boost M1-associated anti-tumor immunity. In this review, we will focus on the potential of macrophages as therapeutic targets and discuss their involvement in state-of-the-art strategies to modulate prevalent pathologies of aging societies.

## Introduction

Macrophages are widely distributed throughout the tissues and display a huge functional heterogeneity. They can acquire pro- or anti-inflammatory functions depending on the surrounding cytokines and tissue microenvironment.

Macrophages have been classified according to a linear scale, on which M1 macrophages represent one extreme and M2 macrophages represent the other.

Macrophage polarization is plastic and reversible. While M1 polarization takes place at the initial stages of the inflammatory response, M2 polarization is predominant during resolution of inflammation. The sequential occurrence of both polarization states is an absolute requirement for the appropriate termination of inflammatory responses, as well as for adequate tissue repair after injury, and alterations in the shift between macrophage polarization states result in chronic inflammatory pathologies, autoimmune diseases, and even metabolic disorders (Murray et al., 2014; Robert A Harris, 2015; Ginhoux et al., 2016).

A main function described for macrophages is their capacity to differentiate between benign and harmful microorganisms through a pathogen-associated molecular pattern (Mogensen, 2009). When they encounter a virus or bacteria, they engulf and destroy it. However, some pathogens subvert this process and instead live inside the macrophage. This provides an environment in which the pathogen is hidden from the immune system and allows it to replicate. Diseases with this type of behavior include tuberculosis (caused by *Mycobacterium* tuberculosis) and leishmaniasis (caused by *Leishmania* species) (Chai et al., 2018; Rossi and Fasel, 2018). In order to minimize the possibility of becoming the host of an intracellular bacteria, macrophages have evolved defense mechanisms such as induction of nitric oxide and reactive oxygen intermediates, which are toxic to microbes, restrict the microbe’s nutrient supply, and induce autophagy (Weiss and Schaible, 2015).

Another dimension described as essential for macrophage polarization is their metabolic profile (Galván-Peña and O’Neill, 2014). Briefly, the metabolism of M1 macrophages is characterized by enhanced glycolysis, flux through the pentose phosphate pathway (PPP), and a truncated TCA cycle, leading to the accumulation of succinate and citrate. Furthermore, the metabolic profile of M2 macrophages is defined by oxidative phosphorylation (OXPHOS), enhanced fatty acid oxidation (FAO) pathway, and a decreased glycolysis and PPP (Mills and O’Neill, 2016; Geeraerts et al., 2017).

Macrophage polarization occurs both in physiological conditions and in pathology. In fact, these polarization stages are considered a key determinant of disease development and/or regression (Sica et al., 2015). Therefore, dissection of the molecular basis of functional macrophage subtypes should allow the identification of molecules, signaling pathways, and metabolic routes which ultimately determine the acquisition of macrophage effector functions under homeostatic and pathological conditions.

Likewise, anti-inflammatory therapies targeting macrophages by specific ablation have been used since a long time ago, displaying relevant efficacy in rheumatoid arthritis (RA), atherosclerosis, vascular injury, and cancer. However, in some cases, significant depletion of macrophages has been associated with immunosuppression, infection, and reduced wound healing (Patel and Janjic, 2015). Thus, it seems reasonable that the next generation of macrophage-based therapies will aim to repolarize macrophages instead of eliminating them. That is the case of several tumor-associated macrophage (TAM)-targeted therapies that include inhibiting macrophage effector functions or reprogramming towards an anti-tumorigenic phenotype, with varying degrees of efficacy (Quail and Joyce, 2017).

In this review, we will focus on specific macrophage populations, aiming to describe their biology and identify potential therapeutic targets useful in the treatment of highly prevalent pathologies such as cancer, RA, and osteoporosis.

## Osteomacs, a Novel Therapeutic Target in Osteoporosis

Osteoporosis is a chronic bone disease characterized by an increased risk of fracture due to the degradation of bone tissue (resorption) by overactivated monocyte-derived osteoclasts, being a leading cause of mortality in the elderly (Cosman et al., 2014). Bone contains different monocyte-derived populations that perform critical functions in skeletal homeostasis (Sinder et al., 2015), including resorption by osteoclasts and regulation of osteoclast actions by cytokine-secreting macrophages. Even though bone anti-resorptive therapies target osteoclasts, other monocyte-derived subpopulations, including osteal macrophages (also named osteomacs), have recently been pointed to play a key role in bone homeostasis (Sinder et al., 2015).

Osteomacs are a population of osteoblast-supportive resident macrophages distributed within bone surfaces that regulate osteoblast-dependent matrix mineralization *in vitro* (Chang et al., 2008). *In vivo*, macrophage ablation in a MaFIA model (macrophage Fas-induced apoptosis transgenic mice, which have an inducible Fas apoptotic system driven by the mouse Csf1 receptor promoter) caused an osteopenic (low bone mass) phenotype with the osteoclastic cell number/activity unchanged, indicating that bone mass reduction was due to a decrease in macrophage-dependent bone formation (van Rooijen et al., 2014).

Different approaches to potentiate macrophage osteogenic actions have been suggested to ameliorate osteoporosis, including those of immunomodulation. Interleukin-4 treatment of M1-polarized macrophage and osteoblast co-cultures showed enhanced osteogenesis by inducing macrophage phenotype shift to M2 (Loi et al., 2016). Similarly, clodronate liposome administration causes high bone mass associated with an increase of M2 osteomacs in a mouse model (van Rooijen et al., 2014). In addition, parathormone (PTH), one of the current therapies to achieving bone formation (Haas and LeBoff, 2018), is also related to macrophage modulation in bone (Chang et al., 2008; Koh et al., 2011; van Rooijen et al., 2014). In the MaFIA model, PTH treatment did not induce bone formation, whereas PTH treatment in control mice increased macrophage presence in bone (Chang et al., 2008). Another model that allows a transient ablation of efferocytic cells (phagocytic macrophages) by clodronate liposome administration showed a greater effect of PTH in bone mass (van Rooijen et al., 2014). This effect was explained by the fact that, after transient macrophage depletion, a rebound of CD68^+^ M2-like macrophages occurs, increasing the secretion of osteogenic factors that might potentiate PTH bone formation (van Rooijen et al., 2014). Based on these evidences, development of therapeutic agents that potentiate M2 macrophage populations in bone might be useful to improve osteoporosis clinical outcome.

Regarding macrophage modulation of bone fracture regeneration, an osteomac–osteoblast association has also been observed in a model of intramembranous fracture healing (Alexander et al., 2011) and during endochondral ossification in a model of bone injury (Alexander et al., 2017). In fact, osteomacs are distributed throughout the bone injury sites in the different stages of bone healing (Alexander et al., 2011). In this regard, a pivotal role in collagen deposition and matrix mineralization during intramembranous bone healing was observed in CSF-1-activated osteomacs (Alexander et al., 2011). Interestingly, preclinical studies show that CSF-1 specifically increases injury-associated osteomacs, but not inflammatory macrophages or osteoclasts, enhancing bone healing (Sarahrudi et al., 2009; Alexander et al., 2011; Hume and MacDonald, 2012; Raggatt et al., 2014). These data suggest the potential key role of CSF-1-activated osteomacs in bone regeneration. Other molecules that modulate inflammatory processes, such as sphingosine 1-phosphate (S1P) (Yang et al., 2018), might modulate macrophage actions during bone repair. Indeed, enhanced bone regeneration using biodegradable polymer scaffolds that deliver S1P receptor-targeted drugs has been observed in cranial defects (Das et al., 2014).

Collectively, these data highlight osteomacs as a novel target in bone physiopathology and suggest that factors involved in their regulation might be a future alternative to broaden the spectrum for osteoporosis treatment and, possibly, for other metabolic bone diseases.

## Targeting Macrophages in Inflammatory Diseases: Rheumatoid Arthritis

**Figure 1:**
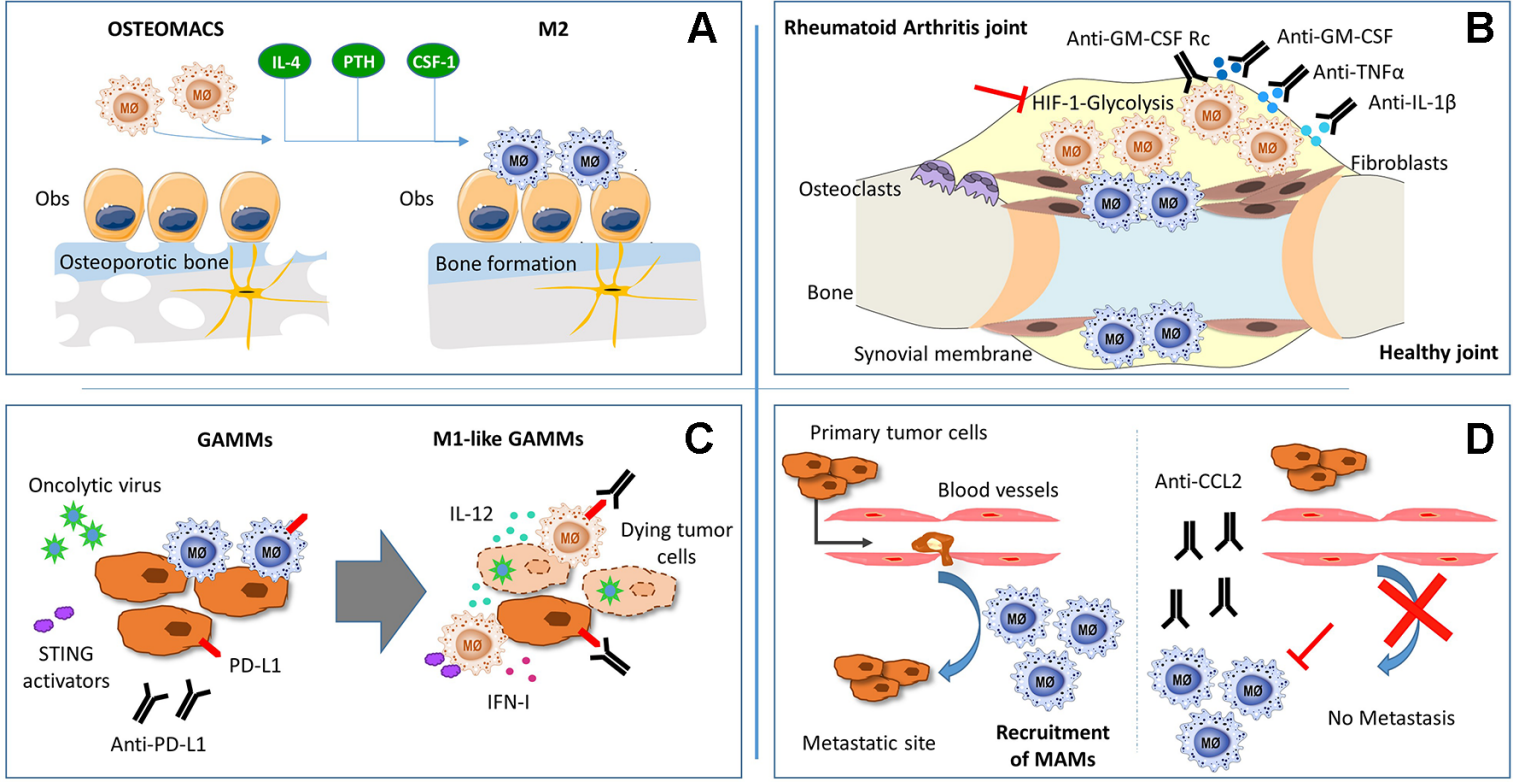
Schematic representation of macrophages function and potential theraputic targets in: **(A)** osteoporosis, **(B)** rheumathoid arthritis **(C)** glioma and **(D)** metastatic tumors.

RA is a chronic inflammatory autoimmune disorder that affects synovial joints. Current therapies may decrease symptom severity and delay progression, but RA is not curable yet. Macrophages are the most numerous immune cells found in the RA synovium, where they produce the predominant pro-inflammatory cytokines involved in RA pathogenesis (TNFα, IL-1β, and IL-6), together with chemoattractant factors (CCL2 and IL-8) and metalloproteinases (MMP-3 and MMP-12) (Kinne et al., 2007). Furthermore, mouse experimental models have confirmed the key role of macrophages in RA pathogenesis (Davignon et al., 2013). Taking into account the effectiveness of the current RA therapies against the main products of activated pro-inflammatory macrophages (TNFα, IL-1β, and IL-6) and the positive correlation between macrophage numbers and joint erosion (Hamilton and Tak, 2009), we can strongly identify macrophages as a crucial target for therapeutic intervention (**Figure 1**).

Due to the plasticity of monocytes and macrophages, pathophysiological stimuli can alter the phenotype and function of resident synovial macrophages as well as induce the migration and differentiation of peripheral monocytes into the synovial joint. Studies in mice and humans suggest that RA synovial macrophages (RA-SM) differentiate from blood monocytes that migrate into the synovium (Herenius et al., 2011; Misharin et al., 2014) and express a specific set of markers (**Table 1**). Actually, in active RA, there is an increase in the total number of macrophages in the synovial membrane, concomitant with a higher expression of pro-inflammatory markers (Smith et al., 2003; Palacios et al., 2015). Interestingly, healthy synovial macrophages express lower levels of M1-associated markers when compared with RA-SM (Smith et al., 2003; Palacios et al., 2015), but display phenotypic features of M2 anti-inflammatory macrophages (Kurowska-Stolarska and Alivernini, 2017) (**Table 1**). Besides this, the RA synovial hypoxic milieu could contribute to the M1 macrophage polarization phenotype of RA-SM (Izquierdo et al., 2015). Therefore, we can assume that macrophage populations are switched towards a pro-inflammatory phenotype in RA environment, and re-polarization of RA-SM could contribute to restore synovium homeostasis (**Figure 1**). In line with this hypothesis, diverse studies have been performed. Particularly, the therapeutic effect of blocking granulocyte-macrophage colony-stimulating factor (GM-CSF) has been confirmed in both RA mouse models (Avci et al., 2016) and in phase I and II clinical trials with RA patients (Behrens et al., 2015; Weinblatt et al., 2018). This cytokine plays a main role in RA since it can be detected in the blood, bone marrow, and synovial fluid of RA patients (Wicks and Roberts, 2016) and represents a well-known M1 polarization inducer (Fleetwood et al., 2014). Notably, it has been shown that RA-SM display a GM-CSF-associated pro-inflammatory profile (Palacios et al., 2015). Another example of a modulator of macrophage polarization with a positive effect in RA is CP-25, a compound derived from total glucosides of peony, which is widely used for the treatment of RA in China (Chang et al., 2016). Thus, there is a correlation between the improvement in RA pathology and a polarization switch towards an M2 anti-inflammatory phenotype. However, the specific activation mechanisms of synovial macrophages remain to be elucidated.

**Table 1:** Phenotypic markers of specific macrophage subpopulations in different pathologies.

Macrophage subpopulations	Markers
**Osteomacs**	F4/80^+^, CD115^+^, Mac-3^+^, CD68^+^, Mac-2/galectin-3^low^, TRAP^low1,2^
**Rheumatoid arthritis-synovial macrophages (RA-SM) and monocytes**	CD50^high^, CD36^high^, MMP12^high^, CCR2^high^, PHD3^high^ (human)CD209^low^, Folate Receptor-^ßlow^(human)^3^
**Glioma-associated macrophages and microglia (GAMMs)**	Iba1^+^, CD11b^+^, CX3CR1^+^, CCR2^−^, Sall1^+^, CD45^low 4^ [microglia] CD11b^+^, CD45^high^, MHCII^high^, CCR2^+5^ [monocyte-derived macrophages]
**Tumor-associated macrophages (TAMs)**	LY6C^+^, MHC-II^+^, CX3CR1^+^, CCR2^+^, I-selectin^+^, TIE2^+6-8^ CD14^+^, CD312^+^, CD115^+^, CD16^+^ (human)
**Metastasis-associated macrophages (MAMs)**	F4/80^+^, CD11b^+^, VEGFR^high^, CCR2^high^ CD11c-^low^ ^6–9^

Table shows macrophage markers in mice, unless otherwise noted. ^1^ Alexander et al. (2011) ^2^ Chang et al. (2008) ^3^ Palacios et al. (2015) ^4^ Buttgereit et al. (2016) ^5^ Saederup et al. (2010) ^6^ Doak et al. (2018) ^7^ Qian et al. (2011) ^8^ Ruffell et al. (2012) ^9^ Rippaus et al. (2016)

Since macrophages are natural phagocytes, a promising future approach is the use of extracellular vesicles (EVs), cell-derived membranous structures that allow intercellular communication. As proof of concept, EVs derived from mesenchymal cells displayed an immunosuppressive effect in RA by inducing an M2 polarization shift (Cosenza et al., 2017; Lo Sicco et al., 2017). Moreover, EV cargo can be manipulated by packing them with specific proteins or miRNA that could skew macrophages towards a homeostatic phenotype (Cosenza et al., 2017). Altogether, we suggest the modulation of macrophage activation as a promising therapeutic approach for RA (**Figure 1**), but first, deeper understanding of macrophage phenotypic heterogeneity and function in the synovial joints must be achieved.

## Immunotherapeutic Strategies Involving Glioma-Associated Microglia and Macrophages

Glioblastoma (GBM, WHO grade IV) is the most aggressive primary brain tumor in adults with poor clinical outcomes despite the current standard of care. Macrophages and microglia—resident brain macrophages originated in the yolk sac during embryogenesis and with expression of specific markers (**Table 1**)—are predominant immune cells in GBM, representing 30–50% of the tumor bulk, which makes them a possible target for treatment (**Table 1**). Both M1 and M2 macrophages have been described in GBM, with M2 correlating with increased tumor growth and worse patient outcome (Carvalho da Fonseca and Badie, 2013). Currently, reprogramming M2 macrophages to anti-tumorigenic M1 is being extensively studied as a strategy for GBM immunotherapy (**Figure 1**).

Blockage of tumor-derived CD47 has been shown to promote M1 polarization, leading to improved tumor phagocytosis (Zhang et al., 2016; Zhu et al., 2017). Although anti-VEGF (vascular endothelial growth factor) therapy with bevacizumab failed in GBM patients, its combination with angiopoietin-2 (Ang-2) inhibition led to M2-to-M1 reprogramming in the tumor microenvironment and prolonged survival in glioma-bearing mice (Yu et al., 2016). Similarly, anti-CSF-1R (colony-stimulating factor 1 receptor) treatment with Pexidartinib (PLX3397) specifically decreased M2 tumor-promoting macrophages (Pyonteck et al., 2013), without benefitting GBM patients (Butowski et al., 2016). Nonetheless, its combination with IGF-1R (insulin-like growth factor 1 receptor) or PI3K (phosphatidylinositol 3-kinase) blockade has given promising results (Quail et al., 2016). Finally, an increased M1/M2 ratio in glioma-associated microglia and macrophages (GAMMs) has been achieved through pharmacological blockade of chemokine receptor CCR5 (Laudati et al., 2017) or neuropilin-1, an M2-associated cell surface receptor with pro-tumorigenic functions (Miyauchi et al., 2016).

So far, classical immunotherapeutic strategies based on immune checkpoint inhibitors have shown limited efficacy in GBM patients. However, oncolytic viruses emerged as promising therapeutics for several types of cancer, not only as lytic agents that kill tumor cells but also as carriers of immunostimulatory molecules that activate durable anti-tumoral immunity. Oncolytic viruses strongly induce IFN-β, one of the key cytokines needed for CD8-mediated tumor rejection, DC maturation, and maintenance of M1 macrophage polarization (Zamarin et al., 2014; Rackov et al., 2016). Recently, oncolytic herpes simplex virus (oHSV) genetically engineered to produce IL-12 (Cheema et al., 2013) and to specifically target tumor cells was shown to induce a remarkable improvement in a murine GBM model (Alessandrini et al., 2019). Moreover, the triple-combination approach using IL-12-producing oHSV together with anti-CTLA-4 and anti-PD-1 antibodies showed a synergistic effect in inducing M1 polarization in TAMs and microglia, leading to glioma eradication in mice (Saha et al., 2017). The anti-tumor effects of immune checkpoint inhibitors are highly dependent on type I interferon and might also be achieved through the activation of STING (stimulator of interferon genes) (Wang et al., 2017). In accordance with this, the STING activator alpha-mangostin has the ability to induce M2-to-M1 macrophage repolarization and exert anti-tumor effects (Zhang et al., 2018).

Metabolic rewiring during microglia and macrophage polarization is currently extensively studied as a target for treatment not only in GBM but also other pathologies in which microglia play critical roles, including neuroinflammatory and neurodegenerative disorders. Although significant challenges remain to be resolved, e.g., the selectivity of such treatment, “metabolic therapeutics” that interfere with key metabolic pathways and modulate macrophage and microglia polarization hold considerable promise for the development of novel combination therapies for GBM. Most likely, the combination of multiple strategies, including metabolites, oncolytic viruses, immunomodulatory cytokines, and checkpoint inhibitors, will need to be clinically explored.

## Targeting Metastasis-Associated Macrophages in Cancer

Metastasis involves the dissemination of cancer cells from primary tumors into surrounding tissues, causing about 90% of cancer-associated deaths (Lambert et al., 2017). Metastasis requires multiple steps, including the participation of TAMs from the primary tumor in the metastatic progression (Doak et al., 2018). TAMs trigger tumor growth, initiate angiogenesis, are required for tumor cell migration and invasion (Qian and Pollard, 2010), and display a specific set of markers (**Table 1**).

Interestingly, recent studies have demonstrated the ability of a distinct population of macrophages to promote secondary tumor development. This population of metastasis-associated macrophages (MAMs) differs in its origin compared to TAMs (Qian et al., 2011; Argyle and Kitamura, 2018). Resident macrophages are preferentially recruited in primary tumors, generating TAMs, whereas inflammatory macrophages are recruited in metastatic sites, generating MAMs (Qian et al., 2011; Kitamura et al., 2015). Moreover, a metastatic site-specific polarization of macrophages has also been reported in intracranial breast cancer metastases (Rippaus et al., 2016). MAMs in dural metastases show a higher antigen-presenting capacity compared to MAMs within parenchymal brain metastases that show a shift towards an M2 state. MAM polarization was directly linked to inflammation-related molecular pathways and expression of cytokines that cancer cells acquire upon site-specific metastasis. These findings are crucial for the development of improved therapies for metastatic disease dependent of MAM phenotypes at different metastatic locations (Rippaus et al., 2016).

Prometastatic changes in organs where metastases later appear have also been described (Kaplan et al., 2007). Such changes induce the formation of “premetastatic niches”, favoring the implantation of circulating tumor cells with an organomimetic phenotype, which satisfies the functional requirements of the niche (Kaplan et al., 2007). That is the case in metastatic melanoma, were changes in the lung microenvironment were detected prior to the establishment of the metastasis (Kaplan et al., 2005). In this regard, resident macrophages and the recruitment of bone marrow-derived macrophages (BMDMs) induce an inflammatory environment in metastatic sites favoring tumor cell colonization and recruitment of additional BMDMs. The increased BMDM population triggers protumoral functions, promoting extravasation and survival of metastasizing cancer cells (Kitamura et al., 2018). BMDM-derived MAMs interact with cells like endothelial cells or cancer-associated fibroblasts, enhancing the metastatic potential of cancer cells, i.e., increasing endothelial affinity and permeability, which supports circulating cancer cell adhesion and migration into the tissue (Doak et al., 2018). Thus, the cross-communication between tumor cells and cells from the metastatic microenvironment may favor the development of metastatic niches (Ardura et al., 2018).

In fact, the blockage of these interactions has already been used as a therapeutic strategy. Treatment with neutralizing antibodies against CCL2 secreted from both tumor cells and stromal cells inhibited the recruitment of circulating monocytes and subsequent accumulation of MAMs (Qian et al., 2011). In addition, activation of the CCL2–CCR2 axis increases CCL3 secretion from MAMs, resulting in enhanced MAM–cancer cell interaction and prolonged retention of MAMs at the metastases (Kitamura et al., 2015). Moreover, interruption of CCL2 inhibitory treatment led to increased metastases and mortality in four mouse models of metastatic breast cancer (Bonapace et al., 2014) (**Figure 1**).

Other anti-metastatic therapeutic strategies targeting MAMs are based on a cell-permeable peptide derived from caveolin-1. This molecule has already been described as an anti-metastatic regulator of MAMs in a mouse model of lung metastases. In MAMs of this model, Caveolin-1 specifically inhibits vascular endothelial growth factor A/vascular endothelial growth factor receptor 1 (VEGF-A/VEGFR1) signaling and its downstream effectors, matrix metallopeptidase 9 (MMP9) and CSF-1, being critical for metastasis development and progression and not for primary tumor growth (Celus et al., 2017). Even though these results seem promising, further investigation is required to better understand how MAMs regulate metastases to develop future MAM-based therapies (**Figure 1**).

## Conclusions and Future Directions

Here, we present evidences for macrophage polarization in the context of M1-associated inflammatory diseases, such as RA, and M2-related disorders, like cancer and non-inflammatory pathologies represented by osteoporosis. Moreover, we also include recently identified macrophage populations, MAMs and osteomacs, representative of the plasticity of these cells.

We believe that targeting macrophage polarization might lead to novel intervention strategies.

Current approaches using macrophages as therapies have essentially been developed in preclinical mouse models mainly for AR and cancer uses. Even though some seem promising, a few drawbacks need to be overcome to ensure a successful implementation. First, neither murine macrophage nor monocyte-derived macrophages fully represent what occurs in human macrophages during homeostasis or disease. Novel human induced pluripotent stem cell (IPCS)-derived macrophage models have been reported and might contribute to a better knowledge of macrophage polarization biology. Second, limitations of therapies using macrophage education include the specificity and durability of treatment. Thus, targeting a specific subset of macrophages in a particular tissue and avoiding deleterious side effects or reeducation when therapies are discontinued are major problems that also need to be addressed. In fact, a major problem in these therapies is the pathological consequences of repolarization promoting autoimmune or inflammatory diseases.

Improving our understanding of the basic mechanisms underlying macrophage plasticity and the identification of relevant pathological and physiological macrophage populations, in health and diseases, will lead to the development of new molecular tools to achieve the aforementioned challenges.

## Author Contributions

All authors discussed, wrote, and reviewed the manuscript, and designed and created the figure.

## Conflict of Interest

The authors declare that the research was conducted in the absence of any commercial or financial relationships that could be construed as a potential conflict of interest.

## References

[B1] Alessandrini F. Menotti L. Avitabile E. Appolloni I. Ceresa D. Marubbi D. (2019). Eradication of glioblastoma by immuno-virotherapy with a retargeted oncolytic HSV in a preclinical model. Oncogene 38, 4467–4479. 10.1038/s41388-019-0737-2 30755732

[B2] Alexander K. A. Chang M. K. Maylin E. R. Kohler T. Müller R. Wu A. C. (2011). Osteal macrophages promote *in vivo* intramembranous bone healing in a mouse tibial injury model. J. Bone Miner. Res. 26, 1517–1532. 10.1002/jbmr.354 21305607

[B3] Alexander K. A. Raggatt L. J. Millard S. Batoon L. Chiu-Ku Wu A. Chang M. K. (2017). Resting and injury-induced inflamed periosteum contain multiple macrophage subsets that are located at sites of bone growth and regeneration. Immunol. Cell Biol. 95, 7–16. 10.1038/icb.2016.74 27553584

[B4] Ardura J. A. Gutiérrez Rojas I. Álvarez Carrión L. Friedman P. A. Alonso V. (2018). Factors secreted by bone cells induce intracellular calcium accumulation and cyclic AMP and activation of ERK 1/2 in prostate cancer cells; evaluation by fluorescence techniques in living cells. Rev. Osteoporos. y Metab. Miner. 10, 131–138. 10.4321/S1889-836X2018000400005

[B5] Argyle D. Kitamura T. (2018). Targeting macrophage-recruiting chemokines as a novel therapeutic strategy to prevent the progression of solid tumors. Front. Immunol. 9, 2629. 10.3389/fimmu.2018.02629 30483271 PMC6243037

[B6] Avci A. B. Feist E. Burmester G. R. (2016). Targeting GM-CSF in rheumatoid arthritis. Clin. Exp. Rheumatol. 34, 39–44. 27586802

[B7] Behrens F. Tak P. P. Østergaard M. Stoilov R. Wiland P. Huizinga T. W. (2015). MOR103, a human monoclonal antibody to granulocyte - Macrophage colony-stimulating factor, in the treatment of patients with moderate rheumatoid arthritis: results of a phase Ib/IIa randomised, double-blind, placebo-controlled, dose-escalation trial. Ann. Rheum. Dis. 74, 1058–1064. 10.1136/annrheumdis-2013-204816 24534756 PMC4431325

[B8] Bonapace L. Coissieux M.-M. Wyckoff J. Mertz K. D. Varga Z. Junt T. (2014). Cessation of CCL2 inhibition accelerates breast cancer metastasis by promoting angiogenesis. Nature 515, 130–133. 10.1038/nature13862 25337873

[B9] Butowski N. Colman H. De Groot J. F. Omuro A. M. Nayak L. Wen P. Y. (2016). Orally administered colony stimulating factor 1 receptor inhibitor PLX3397 in recurrent glioblastoma: an Ivy Foundation early phase clinical trials consortium phase II study. Neuro. Oncol. 18, 557–564. 10.1093/neuonc/nov245 26449250 PMC4799682

[B10] Buttgereit A. Lelios I. Yu X. Vrohlings M. Krakoski N. R. Gautier E. L. (2016). Sall1 is a transcriptional regulator defining microglia identity and function. Nat. Immunol. 17, 1397–1406. 10.1038/ni.3585 27776109

[B11] Carvalho da Fonseca A. C. Badie B. (2013). Microglia and macrophages in malignant gliomas: recent discoveries and implications for promising therapies. Clin. Dev. Immunol. 2013, 1–5. 10.1155/2013/264124 PMC3707269 23864876

[B12] Celus W. Di Conza G. Oliveira A. I. Ehling M. Costa B. M. Wenes M. (2017). Loss of caveolin-1 in metastasis-associated macrophages drives lung metastatic growth through increased angiogenesis. Cell Rep. 21, 2842–2854. 10.1016/j.celrep.2017.11.034 29212030 PMC5732321

[B13] Chai Q. Zhang Y. Liu C. H. (2018). Mycobacterium tuberculosis: an adaptable pathogen associated with multiple human diseases. Front. Cell. Infect. Microbiol. 8, 158. 10.3389/fcimb.2018.00158 29868514 PMC5962710

[B14] Chang M. K. Raggatt L. Alexander K. A. Kuliwaba J. S. Fazzalari N. L. Schroder K. (2008). Osteal tissue macrophages are intercalated throughout human and mouse bone lining tissues and regulate osteoblast function in vitro and in vivo. J. Immunol. 181, 1232–1244. 10.4049/jimmunol.181.2.1232 18606677

[B15] Chang Y. Jia X. Wei F. Wang C. Sun X. Xu S. (2016). CP-25, a novel compound, protects against autoimmune arthritis by modulating immune mediators of inflammation and bone damage. Sci. Rep. 6, 26239. 10.1038/srep26239 27184722 PMC4869037

[B16] Cheema T. Wakimoto H. Fecci P. E. Ning J. Kuroda T. Jeyaretna D. S. (2013). Multifaceted oncolytic virus therapy for glioblastoma in an immunocompetent cancer stem cell model. Proc. Natl. Acad. Sci. U. S. A. 110, 12006–12011. 10.1073/pnas.1307935110 23754388 PMC3718117

[B17] Cosenza S. Ruiz M. Maumus M. Jorgensen C. Noël D. (2017). Pathogenic or therapeutic extracellular vesicles in rheumatic diseases: role of mesenchymal stem cell-derived vesicles. Int. J. Mol. Sci. 18, E889. 10.3390/ijms18040889 PMC5412468 28441721

[B18] Cosman F. de Beur S. J. LeBoff M. S. Lewiecki E. M. Tanner B. Randall S. (2014). Clinician’s Guide to Prevention and Treatment of Osteoporosis. Osteoporos. Int. 25, 2359–2381. 10.1007/s00198-014-2794-2 25182228 PMC4176573

[B19] Das A. Tanner S. Barker D. A. Green D. Botchwey E. A. (2014). Delivery of S1P receptor-targeted drugs *via* biodegradable polymer scaffolds enhances bone regeneration in a critical size cranial defect. J. Biomed. Mater. Res. - Part A 102, 1210–1218. 10.1002/jbm.a.34779 PMC3951302 23640833

[B20] Davignon J. L. Hayder M. Baron M. Boyer J. F. Constantin A. Apparailly F. (2013). Targeting monocytes/macrophages in the treatment of rheumatoid arthritis. Rheumatol. (United Kingdom) 52, 590–598. 10.1093/rheumatology/kes304 23204551

[B21] Doak G. R. Schwertfeger K. L. Wood D. K., (2018). Distant relations: macrophage functions in the metastatic niche. Trends in Cancer 4, 445–459. 10.1016/j.trecan.2018.03.011 29860988 PMC5990045

[B22] Fleetwood A. J. Lawrence T. Hamilton J. A. Cook A. D. (2014). Granulocyte-macrophage colony-stimulating factor (CSF) and macrophage CSF-dependent macrophage phenotypes display differences in cytokine profiles and transcription factor activities: implications for CSF blockade in inflammation. J. Immunol. 178, 5245–5252. 10.4049/jimmunol.178.8.5245 17404308

[B23] Galván-Peña S. O’Neill L. A. J. (2014). Metabolic reprograming in macrophage polarization. Front. Immunol. 5, 420. 10.3389/fimmu.2014.00420 25228902 PMC4151090

[B24] Geeraerts X. Bolli E. Fendt S. M. Van Ginderachter J. A. (2017). Macrophage metabolism as therapeutic target for cancer, atherosclerosis, and obesity. Front. Immunol. 8, 289. 10.3389/fimmu.2017.00289 28360914 PMC5350105

[B25] Ginhoux F. Schultze J. L. Murray P. J. Ochando J. Biswas S. K. (2016). New insights into the multidimensional concept of macrophage ontogeny, activation and function. Nat. Immunol. 17, 34–40. 10.1038/ni.3324 26681460

[B26] Haas A. V. LeBoff M. S. (2018). Osteoanabolic Agents for Osteoporosis. J. Endocr. Soc. 2, 922–932. 10.1210/js.2018-00118 30087947 PMC6065487

[B27] Hamilton J. A. Tak P. P. (2009). The dynamics of macrophage lineage populations in inflammatory and autoimmune diseases. Arthritis Rheum. 60, 1210–1221. 10.1002/art.24505 19404968

[B28] Herenius M. M. J. Thurlings R. M. Wijbrandts C. A. Bennink R. J. Dohmen S. E. Voermans C. (2011). Monocyte migration to the synovium in rheumatoid arthritis patients treated with adalimumab. Ann. Rheum. Dis. 70, 1160 LP–1162. 10.1136/ard.2010.141549 PMC3086080 21345816

[B29] Hume D. A. MacDonald K. P. A. (2012). Therapeutic applications of macrophage colony-stimulating factor-1 (CSF-1) and antagonists of CSF-1 receptor (CSF-1R) signaling. Blood 119, 1810–1820. 10.1182/blood-2011-09-379214 22186992

[B30] Izquierdo E. Cuevas V. D. Fernández-Arroyo S. Riera-Borrull M. Orta-Zavalza E. Joven J. (2015). Reshaping of Human Macrophage Polarization through Modulation of Glucose Catabolic Pathways. J. Immunol. 195, 2442–2451. 10.4049/jimmunol.1403045 26209622

[B31] Kaplan R. N. Psaila B. Lyden D. (2007). Bone marrow cells in the “pre-metastatic niche”: within bone and beyond. Cancer Metastasis Rev. 25, 521–529. 10.1007/s10555-006-9036-9 17186383

[B32] Kaplan R. N. Riba R. D. Zacharoulis S. Bramley A. H. Vincent L. Costa C. (2005). VEGFR-1 positive haematopoietic bone marrow progenitors initiate the pre-metastatic niche. Nature 438, 820–827. 10.1038/nature04186 16341007 PMC2945882

[B33] Kinne R. W. Stuhlmüller B. Burmester G.-R. (2007). Cells of the synovium in rheumatoid arthritis. Macrophages. Arthritis Res. Ther. 9, 224. 10.1186/ar2333 18177511 PMC2246244

[B34] Kitamura T. Doughty-Shenton D. Cassetta L. Fragkogianni S. Brownlie D. Kato Y. (2018). Monocytes differentiate to immune suppressive precursors of metastasis-associated macrophages in mouse models of metastatic breast cancer. Front. Immunol. 8, 17. 10.3389/fimmu.2017.02004 PMC5776392 29387063

[B35] Kitamura T. Qian B.-Z. Soong D. Cassetta L. Noy R. Sugano G. (2015). CCL2-induced chemokine cascade promotes breast cancer metastasis by enhancing retention of metastasis-associated macrophages. J. Exp. Med. 212, 1043–1059. 10.1084/jem.20141836 26056232 PMC4493415

[B36] Koh A. J. Novince C. M. Li X. Wang T. Taichman R. S. McCauley L. K. (2011). An irradiation-altered bone marrow microenvironment impacts anabolic actions of PTH. Endocrinology 152, 4525–4536. 10.1210/en.2011-1515 22045660 PMC3230047

[B37] Kurowska-Stolarska M. Alivernini S. (2017). Synovial tissue macrophages: friend or foe? RMD Open 3, e000527. 10.1136/rmdopen-2017-000527 29299338 PMC5729306

[B38] Lambert A. W. Pattabiraman D. R. Weinberg R. A. (2017). Emerging Biological Principles of Metastasis. Cell 168, 670–691. 10.1016/j.cell.2016.11.037 28187288 PMC5308465

[B39] Laudati E. Curro D. Navarra P. Lisi L. (2017). Blockade of CCR5 receptor prevents M2 microglia phenotype in a microglia-glioma paradigm. Neurochem. Int. 108, 100–108. 10.1016/j.neuint.2017.03.002 28279751

[B40] Loi F. Córdova L. A. Zhang R. Pajarinen J. Lin T. H. Goodman S. B. (2016). The effects of immunomodulation by macrophage subsets on osteogenesis *in vitro.* Stem Cell Res. Ther. 7, 15. 10.1186/s13287-016-0276-5 26801095 PMC4724110

[B41] Lo Sicco C. Reverberi D. Balbi C. Ulivi V. Principi E. Pascucci L. (2017) . Mesenchymal stem cell-derived extracellular vesicles as mediators of anti-inflammatory effects: endorsement of macrophage polarization. Stem Cells Transl. Med. 6, 1018–1028. 10.1002/sctm.16-0363 28186708 PMC5442783

[B42] Mills E. L. O’Neill L. A. (2016). Reprogramming mitochondrial metabolism in macrophages as an anti-inflammatory signal. Eur. J. Immunol. 46, 13–21. 10.1002/eji.201445427 26643360

[B43] Misharin A. V. Cuda C. M. Saber R. Turner J. D. Gierut A. K. Kenneth Haines G. K. (2014). Nonclassical Ly6C- monocytes drive the development of inflammatory arthritis in mice. Cell Rep. 9, 591–604. 10.1016/j.celrep.2014.09.032 25373902 PMC4223808

[B44] Miyauchi J. T. Chen D. Choi M. Nissen J. C. Shroyer K. R. Djordevic S. (2016). Ablation of Neuropilin 1 from glioma-associated microglia and macrophages slows tumor progression. Oncotarget 7, 9801–9814. 10.18632/oncotarget.6877 26755653 PMC4891085

[B45] Mogensen T. H. (2009). Pathogen recognition and inflammatory signaling in innate immune defenses. Clin. Microbiol. Rev. 22, 240–273. 10.1128/CMR.00046-08 19366914 PMC2668232

[B46] Murray P. J. Allen J. E. Biswas S. K. Fisher E. A. Gilroy D. W. Goerdt S. (2014). Macrophage activation and polarization: nomenclature and experimental guidelines. Immunity 41, 14–20. 10.1016/j.immuni.2014.06.008 25035950 PMC4123412

[B47] Palacios B. S. Estrada-Capetillo L. Izquierdo E. Criado G. Nieto C. Municio C. (2015). Macrophages from the synovium of active rheumatoid arthritis exhibit an activin a-dependent pro-inflammatory profile. J. Pathol. 235, 515–526. 10.1002/path.4466 25319955

[B48] Patel S. K. Janjic J. M. (2015). Macrophage targeted theranostics as personalized nanomedicine strategies for inflammatory diseases. Theranostics 5, 150–172. 10.7150/thno.9476 25553105 PMC4279001

[B49] Pyonteck S. M. Akkari L. Schuhmacher A. J. Bowman R. L. Sevenich L. Quail D. F. (2013). CSF-1R inhibition alters macrophage polarization and blocks glioma progression. Nat. Med. 19, 1264–1272. 10.1038/nm.3337 24056773 PMC3840724

[B50] Qian B.-Z. Li J. Zhang H. Kitamura T. Zhang J. Campion L. R. (2011). CCL2 recruits inflammatory monocytes to facilitate breast-tumour metastasis. Nature 475, 222–225. 10.1038/nature10138 21654748 PMC3208506

[B51] Qian B. Z. Pollard J. W. (2010). Macrophage diversity enhances tumor progression and metastasis. Cell 141, 39–51. 10.1016/j.cell.2010.03.014 20371344 PMC4994190

[B52] Quail D. F. Bowman R. L. Akkari L. Quick M. L. Schuhmacher A. J. Huse J. T. (2016). The tumor microenvironment underlies acquired resistance to CSF1R inhibition in gliomas. Science (80-.) 352, aad3018. 10.1126/science.aad3018 PMC5450629 27199435

[B53] Quail D. F. Joyce J. A. (2017). Molecular pathways: deciphering mechanisms of resistance to macrophage-targeted therapies. Clin. Cancer Res. 23, 876–884. 10.1158/1078-0432.CCR-16-0133 27895033 PMC5453188

[B54] Rackov G. Hernández-Jiménez E. Shokri R. Carmona-Rodríguez L. Mañes S. Álvarez-Mon M. (2016). p21 mediates macrophage reprogramming through regulation of p50-p50 NF- κB and IFN-β. J. Clin. Invest. 126, 3089–3103. 10.1172/JCI83404 27427981 PMC4966310

[B55] Raggatt L. J. Wullschleger M. E. Alexander K. A. Wu A. C. K. Millard S. M. Kaur S. (2014). Fracture healing *via* periosteal callus formation requires macrophages for both initiation and progression of early endochondral ossification. Am. J. Pathol. 184, 3192–3204. 10.1016/j.ajpath.2014.08.017 25285719

[B56] Rippaus N. Taggart D. Williams J. Andreou T. Wurdak H. Wronski K. (2016). Metastatic site-specific polarization of macrophages in intracranial breast cancer metastases. Oncotarget 7, 41473–41487. 10.18632/oncotarget.9445 27203741 PMC5173073

[B57] Robert A Harris C. D. M. (2015). Macrophage polarization: decisions that affect health. J. Clin. Cell. Immunol. 6, 364. 10.4172/2155-9899.1000364 26962469 PMC4780849

[B58] Rossi M. Fasel N. (2018). How to master the host immune system? Leishmania parasites have the solutions! Int. Immunol. 30, 103–111. 10.1093/intimm/dxx075 29294040 PMC5892169

[B59] Ruffell B. Affara N. I. Coussens L. M. (2012). Differential Macrophage Programming in the Tumor Microenvironment Macrophages in solid malignancies. Trends Immunol. 33, 119–126. 10.1016/j.it.2011.12.001 22277903 PMC3294003

[B60] Saederup N. Cardona A. E. Croft K. Mizutani M. Cotleur A. C. Tsou C. L. (2010). Selective chemokine receptor usage by central nervous system myeloid cells in CCR2-red fluorescent protein knock-in mice. PLoS One 5. 10.1371/journal.pone.0013693 PMC2965160 21060874

[B61] Saha D. Martuza R. L. Rabkin S. D. (2017). Macrophage polarization contributes to glioblastoma eradication by combination immunovirotherapy and immune checkpoint blockade. Cancer Cell 32, 253–267.e5. 10.1016/j.ccell.2017.07.006 28810147 PMC5568814

[B62] Sarahrudi K. Mousavi M. Grossschmidt K. Sela N. König F. Vécsei V. (2009). The impact of colony-stimulating factor-1 on fracture healing: an experimental study. J. Orthop. Res. 27, 36–41. 10.1002/jor.20680 18634005

[B63] Sica A. Erreni M. Allavena P. Porta C. (2015). Macrophage polarization in pathology. Cell. Mol. Life Sci. 72, 4111–4126. 10.1007/s00018-015-1995-y 26210152 PMC11113543

[B64] Sinder B. P. Pettit A. R. McCauley L. K. (2015). Macrophages: their emerging roles in bone. J. Bone Miner. Res. 30, 2140–2149. 10.1002/jbmr.2735 26531055 PMC4876707

[B65] Smith M. D. Barg E. Weedon H. Papengelis V. Smeets T. Tak P. P. (2003). Microarchitecture and protective mechanisms in synovial tissue from clinically and arthroscopically normal knee joints. Ann. Rheum. Dis. 62, 303 LP–303307. 10.1136/ard.62.4.303 PMC1754505 12634226

[B66] van Rooijen N. Entezami P. Koh A. J. McCauley L. K. Soki F. N. Park S. I. (2014). Osteal macrophages support physiologic skeletal remodeling and anabolic actions of parathyroid hormone in bone. Proc. Natl. Acad. Sci. 111, 1545–1550. 10.1073/pnas.1315153111 24406853 PMC3910564

[B67] Wang H. Hu S. Shi H. Sun L. Chen Z. J. Chen C. (2017). cGAS is essential for the antitumor effect of immune checkpoint blockade. Proc. Natl. Acad. Sci. 114, 1637–1642. 10.1073/pnas.1621363114 28137885 PMC5320994

[B68] Weinblatt M. E. McInnes I. B. Kremer J. M. Miranda P. Vencovsky J. Guo X. (2018). A Randomized Phase IIb Study of Mavrilimumab and Golimumab in Rheumatoid Arthritis. Arthritis Rheumatol. 70, 49–59. 10.1002/art.40323 28941039 PMC5767745

[B69] Weiss G. Schaible U. E. (2015). Macrophage defense mechanisms against intracellular bacteria. Immunol. Rev. 264, 182–203. 10.1111/imr.12266 25703560 PMC4368383

[B70] Wicks I. P. Roberts A. W. (2016). Targeting GM-CSF in inflammatory diseases. Nat. Rev. Rheumatol. 12, 37–48. 10.1038/nrrheum.2015.161 26633290

[B71] Yang J. Yang L. Tian L. Ji X. Yang L. Li L. (2018). Sphingosine 1-phosphate (S1P)/S1P receptor2/3 axis promotes inflammatory M1 polarization of bone marrow-derived monocyte/macrophage *via* G(α)i/o/PI3K/JNK pathway. Cell. Physiol. Biochem. 49, 1677–1693. 10.1159/000493611 30231248

[B72] Yu V. Munn L. L. Jung K. Riedemann L. Xu L. Batista A. (2016). Ang-2/VEGF bispecific antibody reprograms macrophages and resident microglia to anti-tumor phenotype and prolongs glioblastoma survival. Proc. Natl. Acad. Sci. 113, 4476–4481. 10.1073/pnas.1525360113 27044098 PMC4843473

[B73] Zamarin D. Holmgaard R. B. Subudhi S. K. Park J. S. Mansour M. Palese P. (2014). Localized oncolytic virotherapy overcomes systemic tumor resistance to immune checkpoint blockade immunotherapy. Sci. Transl. Med. 6, 226ra32–226ra32. 10.1126/scitranslmed.3008095 PMC4106918 24598590

[B74] Zhang M. Hutter G. Kahn S. A. Azad T. D. Gholamin S. Xu C. Y. (2016). Anti-CD47 treatment stimulates phagocytosis of glioblastoma by M1 and M2 polarized macrophages and promotes M1 polarized macrophages *in vivo.* PLoS One 11, 1–21. 10.1371/journal.pone.0153550 PMC4836698 27092773

[B75] Zhang Y. Sun Z. Pei J. Luo Q. Zeng X. Li Q. (2018). Identification of α-mangostin as an agonist of human STING. ChemMedChem 13, 2057–2064. 10.1002/cmdc.201800481 30079976

[B76] Zhu H. Leiss L. Yang N. Rygh C. B. Mitra S. S. Cheshier S. H. (2017). Surgical debulking promotes recruitment of macrophages and triggers glioblastoma phagocytosis in combination with CD47 blocking immunotherapy. Oncotarget 8, 12145–12157. 10.18632/oncotarget.14553 28076333 PMC5355332

